# Advances in Intestinal Restoration and Abdominal Wall Reconstruction in Bogotá: A Two-Stage Approach During the Same Hospitalization

**DOI:** 10.3389/jaws.2026.15885

**Published:** 2026-03-16

**Authors:** Juan Pablo Ruiz, Neil Valentín Vega, Alejandro Lora Aguirre, Arnold José Barrios, Angie Carolina Riscanevo Bobadilla, Julián Orrego

**Affiliations:** 1 Abdominal Wall Surgery Group, Department of General Surgery, Clínicas Colsanitas, Bogotá, Colombia; 2 Fundación Universitaria Sanitas, Bogotá, Colombia

**Keywords:** incisional hernia, abdominal wall, intestinal fistula, colostomy, surgical stomas, surgical procedures, ostomy

## Abstract

**Background:**

The available evidence regarding the sequential performance of gastrointestinal tract restoration and abdominal wall reconstruction in two surgical stages during a single hospitalization is limited and is based primarily on case series. In this study, we present our experience with the aim of describing the outcomes obtained in the repair of complex abdominal wall defects and the restoration of intestinal continuity using a two-stage approach within the same hospital stay.

**Methods:**

Case series of patients who underwent elective surgery for gastrointestinal tract restoration, followed by abdominal wall reconstruction in a second surgical stage during the same hospitalization. Medical records of procedures performed between 2018 and 2023 were reviewed. All interventions were carried out electively by a multidisciplinary team involving the abdominal wall surgery group and colorectal surgery.

**Results:**

A total of 30 patients were included. Both surgical procedures were completed in 73% of cases, with a mean interval of 6.3 days between the two surgeries. In 26% of patients, it was not possible to complete both procedures; the most frequent causes were anastomotic leakage and surgical site infection, each occurring in 9% of cases. The mean length of hospital stay was 14 days. The most common complications were postoperative ileus, anastomotic leakage, intestinal perforation, and deep surgical site infection.

**Conclusion:**

Although concomitant surgery is associated with a higher risk of complications particularly in the setting of complex hernias—in appropriately selected patients, sequential procedures performed during the same hospitalization can achieve favorable outcomes, especially in stoma reversal. The implementation of prehabilitation programs and the adoption of shared decision-making models are essential to optimize outcomes and reduce associated morbidity.

## Introduction

Restoration of the gastrointestinal tract in patients with temporary stomas represents a surgical challenge [1], as more than 22% of these patients present with a concomitant incisional hernia at the site of a previous laparotomy, which may coexist with a parastomal hernia, thereby increasing the complexity of the surgical approach [2, 3].

Abdominal wall surgery and restoration of gastrointestinal continuity during the same hospitalization remain a subject of debate, with conflicting clinical evidence [2, 4–6]. This comprehensive approach allows both conditions to be addressed simultaneously—either in a single operative session and/or within the same hospital stay—which may translate into greater efficiency, reduced costs, and improved patient satisfaction by avoiding separate hospitalizations. However, many of these patients require multiple interventions, and the second surgery is frequently not performed, potentially resulting in a disabling hernia or the need for an emergency procedure [7].

Several studies advocate for a staged approach, supported by reported increases in surgical site infection, hernia recurrence, the need for extensive adhesiolysis, and higher rates of anastomotic leakage [7, 8]. In addition, concomitant management has been associated with an increased risk of mesh infection, high technical complexity, and postoperative complications—particularly anastomotic leakage—as well as a possible increase in mortality in complex repairs or high-complexity colorectal procedures [9–11].

In this study, we propose that a simultaneous approach to stoma reversal and incisional hernia repair, performed in patients who undergo preoperative optimization and with the participation of both colorectal surgery and abdominal wall surgery teams, may represent a safe and effective strategy to address both conditions during the same hospitalization, thereby reducing the morbidity associated with sequential procedures.

The objective of this study was to evaluate the clinical outcomes of incisional hernia repair performed concomitantly with stoma closure during a single hospitalization. Complications associated with the combined approach were analyzed as the primary outcome, while length of hospital stay, surgical success rate, and the incidence of reinterventions due to anastomotic leakage were assessed as secondary outcomes.

## Materials and Methods

The study was conducted at Clínica Universitaria Colombia, a university hospital in Bogotá, Colombia. Adult patients who underwent elective gastrointestinal tract restoration and abdominal wall reconstruction performed in sequential two-stage approach during the same hospitalization between July 2018 and December 2023 were included.

Patients who underwent localized stoma-closure approaches requiring postoperative prophylactic mesh placement at the resulting abdominal wall defect were excluded. Patients with complex hernias were included, defined as those associated with lipodystrophy, sequelae of open abdomen, loss of domain, multiple recurrences, parastomal hernias, abdominal wall tumors, or giant hernia defects.

### Therapeutic Strategy

Patients were managed by the institution’s abdominal wall surgery group and were evaluated in a multidisciplinary surgical board to establish and tailor surgical strategies on a case-by-case basis.

Preoperatively, the gastrointestinal tract was evaluated both proximally and distally using endoscopy, along with abdominal computed tomography, which is routinely employed for. Surgical planning in complex abdominal wall surgery. Abdominal wall defects were classified according to the European Hernia Society (EHS) guidelines [12]. In cases with loss of domain of the hernia sac and in accordance with the strategy described by Tanaka et al. (>25%) [13], Patients underwent prehabilitation with botulinum toxin (BTX) and/or progressive preoperative pneumoperitoneum (PPP).

All patients were managed according to the institutional ERAS (Enhanced Recovery After Surgery) protocol as part of their optimization process. This included stabilization of cardiopulmonary disease, smoking cessation, achievement of a body mass index (BMI) <40, and glycated hemoglobin <7.5%. In selected cases, preoperative nutritional supplementation was provided. Multidisciplinary care involved nutritional support, physical therapy, respiratory therapy, psychology, pain management (after the first surgery) and, according to patient needs, internal medicine.

Patients were actively involved in their preoperative optimization through educational sessions as part of the ERAS strategy. The first surgical stage was directed toward gastrointestinal tract restoration, followed by abdominal wall reconstruction during a subsequent surgical stage within the same hospitalization.

All patients received intravenous antibiotic prophylaxis with cefuroxime plus metronidazole, and cefazolin during the second procedure. In patients with penicillin allergy, clindamycin and aztreonam were used. Gastrointestinal surgical procedures included intestinal resection and anastomosis, stoma reversal, and resection of enterocutaneous fistula(e). One or more of the authors participated in all surgeries, and mechanical stapling was used in 100% of the procedures.

### Abdominal Wall Reconstruction Technique

The abdominal wall reconstruction technique was individualized based on defect size, location, and the quality of adjacent tissues. Primary fascial closure without tension was pursued whenever possible, with or without component separation techniques depending on each case.

The majority of patients undergo prosthetic repair in the retromuscular (sublay) position, with a preference for posterior component separation techniques performed through a retromuscular approach. The two most commonly employed techniques are the Rives and Rives–Stoppa methods, due to their well-established benefits, including lower rates of wound complications, reduced prosthetic exposure, and improved long-term functional integration.

However, the size and complexity of certain hernia defects require an individualized approach.

In cases where anatomical and functional restoration of the abdominal wall cannot be achieved despite optimal preoperative optimization and posterior component separation, anterior component separation is employed. While this approach allows repair of larger defects, it is associated with a higher risk of surgical site occurrences (SSO).

Some patients required mesh-mediated traction as a temporary abdominal wall closure strategy following complex intestinal resections, particularly in those at high risk of surgical field contamination or physiological instability. This approach was used as an intermediate stage prior to definitive abdominal wall reconstruction, with the aim of preserving abdominal wall anatomy, preventing lateral muscle retraction, and facilitating subsequent fascial closure under optimal conditions.

**FIGURE 1:**
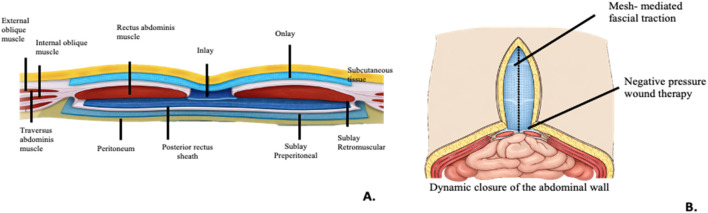
Abdominal wall anatomical planes and prosthetic positioning: illustration of mesh placement and dynamic closure assisted by negative pressure wound therapy. **(A)** Schematic representation of the different anatomical planes of the abdominal wall and the positioning of prosthetic meshes according to the type and location of the defect. **(B)** Application of negative pressure wound therapy with medial fascial traction and dynamic abdominal wall closure.

Mesh-mediated traction consisted of placing a high-density polypropylene (Prolene) mesh in. An inlay position fixed to the aponeurosis, combined with a negative pressure wound therapy system to minimize the defect and prevent lateral retraction of the abdominal muscles Figure 1B.

In cases where a low likelihood of midline closure was anticipated—particularly in patients refractory to abdominal wall prehabilitation strategies (PPP and/or BTX)—the hernia sac was preserved. In these cases, the hernia sac acted as a biological barrier, isolating the intestinal loops from direct contact with the prosthesis and improving prosthetic integration with biological tissue.

### Postoperative Follow-Up and Second Stage Criteria

Postoperative follow-up included close clinical monitoring, with serial assessment of heart rate and overall patient status. Complementary laboratory monitoring included C-reactive protein, leukocyte count, and serum electrolytes on postoperative days 1, 3, and 5.

Tolerance of oral intake and the presence of effective intestinal transit were considered indicators of adequate gastrointestinal recovery. The absence of clinical, biochemical, or functional signs suggestive of anastomotic leakage was mandatory before proceeding to the second intervention.

The second surgical stage was performed during the same hospital admission. The optimal timing was determined on an individualized basis, considering clinical stability, normalization or favorable trends in inflammatory markers, adequate oral intake tolerance, and the presence of intestinal transit, in conjunction with multidisciplinary team assessment.

The preferred technique for incisional hernia repair was the retromuscular approach, combined with posterior component separation (Transversus Abdominis Release, TAR) in hernias with a transverse diameter up to 10 cm. In cases with a transverse diameter greater than 10 cm or when retromuscular repair was not feasible, anterior component separation was performed.

Midline closure was achieved using a small-bites technique with slowly absorbable polydioxanone (PDS) 2/0 sutures. The mesh used was medium-weight, macroporous, monofilament polypropylene, selected at the surgeon’s discretion. In cases with excessive adipocutaneous tissue, a concomitant panniculectomy was performed. When midline closure could not be achieved, a double polypropylene mesh (medium density) was used (inlay + underlay positions) over the hernia sac Figure 1A.

### Statistical Analysis

A retrospective review of a prospectively maintained database was performed, with a descriptive analysis of demographic and clinical characteristics, postoperative outcomes, and associated complications. The study was conducted in accordance with the ethical principles of the Declaration of Helsinki. Informed consent was obtained from all participants, and medical data confidentiality was ensured. The study was approved by the institutional ethics committee (072-24 UNV).

Sociodemographic data collected included age, sex, body mass index, comorbidities, and toxic habits. Data related to the incisional hernia included size and classification according to the European Hernia Society (EHS) [12]. Surgical variables included association with intestinal resections, component separation and its type, number and type of meshes placed, and the surgical technique performed.

Data were analyzed using R statistical software version 4.3.1. Qualitative variables were described using absolute and relative frequencies, while quantitative variables were described using measures of central tendency and dispersion, reporting mean and standard deviation or median and confidence interval, as appropriate.

## Results

During the study period, 30 patients underwent simultaneous repair of complex incisional hernia and intestinal tract reconstruction. The majority of patients were male, accounting for 70% (n = 21) of the cohort, with a mean age of 51.5 years (range: 18–79 years). The sociodemographic characteristics and underlying pathology of the study population are detailed in Table 1.

**TABLE 1:** Demographic and clinical characteristics of the study population.

Variable	Result N: 30
Age, mean (SD), years	52 (17)
Sex, n (%)	
Male	21 (70%)
Female	9 (30%)
Presence of comorbidities, n (%)	19 (63%)
Hypertension	26.7% (n = 8)
Colorectal cancer (colon, cecum, sigmoid, right colon)	20.0% (n = 6)
Ischemic heart disease/atrial fibrillation/AV block	10.0% (n = 3)
Hypothyroidism	13.3% (n = 4)
Obesity	6.7% (n = 2)

SD, Standard deviation. Data are presented as mean (SD) or n (%).

Regarding the type of ostomy, Hartmann’s reversal was performed in 9 patients (30%), followed by ileostomy closure in 9 patients (30%), cecostomy closure in 1 patient (3.3%), and loop colostomy closure in 1 patient (3.3%). The remaining cases were not related to ostomies but to other conditions, most notably enterocutaneous fistula, which was present in 20% of cases (n = 6).

Table 2 describes the characteristics of the hernias. The mean transverse hernia diameter was 12.8 cm, with a range from 5 cm to 20 cm. In 8 cases, abdominal wall prehabilitation strategies were required, with botulinum toxin being the most frequently used, followed by progressive preoperative pneumoperitoneum in 1 case, and combined strategies of progressive preoperative pneumoperitoneum and botulinum toxin in 1 patient.

**TABLE 2:** Surgical characteristics.

Variable	Result N:30
Inter-rectus distance/defect, cm (mean ± SD)	12.8 ± 3.6
Type of ostomy, n (%)	
Hartmann’s procedure	9 (30%)
Ileostomy	9 (30%)
Cecostomy	1 (3.3%)
Loop colostomy	1 (3.3%)
Unknown	10 (33.3%)
Interval between both reconstructions, days (mean ± SD)	6.3 ± 4.2
Prehabilitation strategy, n (%)	
Botulinum toxin	6 (20%)
Progressive preoperative pneumoperitoneum + botulinum toxin	1 (3.3%)
Progressive preoperative pneumoperitoneum	1 (3.3%)
None	22 (73.3%)

SD, Standard deviation.

Anterior component separation was required and represented the most prevalent technique in this study, being used in 56.6% of the cases in which both surgical stages were completed.

When patients were classified according to the European Hernia Society (EHS) ventral hernia classification, the most prevalent category in our cohort was M1M5W3, corresponding to incisional hernias involving the majority of the midline of the abdominal wall (Figure 1).

In this study, both surgical procedures were completed in 73.33% (n = 22) of cases. The time interval between procedures was 6.3 days. Surgical and follow-up characteristics are summarized in Table 2.

In 26.6% of cases (n = 8), abdominal wall reconstruction could not be completed. The most frequent causes were anastomotic dehiscence and surgical site infection, each present in 10% of cases. Other causes included bacteremia and the presence of retracted fascia or poor tissue quality.

**FIGURE 2:**
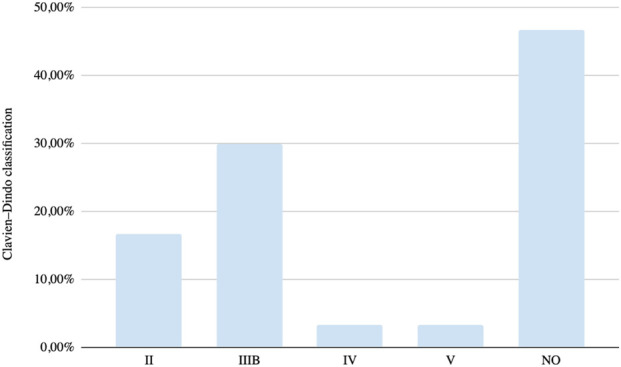
Postoperative complications classified according to the Clavien–Dindo classification system.

In the majority of cases (46.6%), no complications occurred, allowing successful completion of both procedures. According to the Clavien–Dindo classification, 30% of patients experienced grade IIIB complications. The most common complication was postoperative ileus (16%), followed by anastomotic dehiscence and deep surgical site infection, each occurring in 6% of cases. In addition, a mortality rate of 3% (one patient) was reported, secondary to anastomotic dehiscence with subsequent multiple organ dysfunction. Figure 2.

## Discussion

Simultaneous repair of complex incisional hernias and restoration of intestinal continuity remains a subject of debate due to technical complexity and the potential risk of postoperative complications. In this case series, 30 patients with heterogeneous clinical and surgical characteristics were included. The success rate for completing both procedures during the same hospitalization was 73%, a figure comparable to that reported in other similar case series, ranging from 65% to 80% [12, 14–16]. The risk of wound-related complications must be weighed against the risk of hernia recurrence when determining whether definitive abdominal wall reconstruction is appropriate at the time of gastrointestinal reconstruction [13].

The absence of unified protocols for managing patients with incisional hernia and ostomy, together with persistent concerns regarding the risk of infection associated with mesh use in contaminated or potentially contaminated fields, has led many surgeons to favor a staged approach. This strategy aims to reduce infectious complications and optimize local conditions prior to definitive abdominal wall reconstruction. In our cohort, the mean interval between intestinal restoration and abdominal wall reconstruction was 6.3 days, allowing verification of anastomotic integrity and exclusion of leakage prior to the second intervention, while also permitting physiological recovery and thereby reducing the risk of complications during abdominal wall reconstruction. This two-stage strategy within a single hospitalization seeks to balance the benefits of a simultaneous approach—such as reduced costs, fewer hospitalizations, and lower anesthetic risk—with improved control of infectious risk, particularly in contaminated or potentially septic scenarios. Importantly, contamination and surgical site infection may occur in up to 40% of cases, significantly increasing the complexity of both surgical and postoperative management [2].

In our cohort, the main reasons for failure to perform the second procedure were anastomotic dehiscence and surgical site infection, each occurring in up to 10% of cases. These findings are consistent with the literature, where such complications represent major causes of failure of thecombined approach [4, 5]. In line with our results, Rudnicki et al. analyzed a cohort of 62 patients who underwent Hartmann’s reversal with concomitant hernia repair, performed simultaneously in 32 cases and in a second operation in 30 cases. The authors reported a significantly higher rate of perioperative complications in the combined surgery group (57% vs. 20%, p < 0.01). Notably, 25% of these patients developed surgical site infection, wound dehiscence, or repair failure; consequently, two-thirds underwent repair using suture alone without prosthetic mesh [17].

Within the overall complication rate, the most frequent event was postoperative ileus (13.3%), followed by anastomotic dehiscence, deep surgical site infection, and intestinal perforation (each 6.6%). Less common complications included hemoperitoneum, soft tissue necrosis, intestinal obstruction, ureteral injury, and one case of mortality (3.3%; n = 1). When compared with the results reported by Curran et al., who described complication rates of up to 39%–43% in combined procedures, our outcomes suggest a favorable morbidity profile. This difference likely reflects rigorous patient selection, adequate preoperative optimization, and a coordinated approach between colorectal and abdominal wall surgery teams, which enabled anticipation and mitigation of intraoperative risks [3].

In our series, the mean transverse diameter of hernia defects was 12.8 cm, underscoring the complexity of the cases included. Given these dimensions, anterior component separation was performed in 68% of patients and posterior component separation in 6.6%, with the aim of facilitating tension-free fascial closure and optimizing reconstruction of the muscular plane.

In addition, prehabilitation strategies were implemented in 26% of patients, including botulinum toxin injection and, to a lesser extent, progressive preoperative pneumoperitoneum.

In most cases (20%), botulinum toxin was used alone, while 3.3% received combined therapy and another 3.3% underwent pneumoperitoneum alone. These interventions proved particularly useful in patients with large defects, enabling complete fascial closure in 100% of cases.

Our findings are consistent with those reported by Carles Olona et al., who applied botulinum toxin in patients with hernias with transverse diameters greater than 12 cm and similarly achieved complete fascial closure in all treated cases. According to the authors, toxin-induced muscle relaxation facilitates aponeurotic approximation and reduces intra-abdominal pressure, thereby improving repair quality and decreasing tension along the midline [18].

Appropriate patient selection remains the key determinant of success for this approach. We propose a protocol for simultaneous procedures during the same hospitalization that emphasizes preoperative optimization, including enrollment in the ERAS protocol, preoperative weight reduction, and glycemic control to reduce obesity-related morbidity. Smoking cessation was also strongly encouraged. All cases were discussed in a multidisciplinary surgical board to individualize surgical strategies, involving collaboration between surgeons with expertise in colorectal and abdominal wall pathology, thereby minimizing factors associated with morbidity in this patient population [19, 20].

Overall, our results support the feasibility of a sequential approach during the same hospitalization in selected patients, particularly those undergoing ostomy reversal. The implementation of prehabilitation protocols and shared decision-making between colorectal and abdominal wall surgery teams is essential to optimize outcomes and reduce complications.

Regarding study limitations, first, this is a relatively small case series from a single center.

While this limits generalizability, it allowed for control of procedural conditions and ensured participation of a consistent surgical team with expertise in each specialty. Another limitation is the retrospective design, although the study is based on a prospectively maintained database.

The complexity of the cases hindered standardization of surgical techniques and precluded a randomized prospective study; thus, we believe these patients require collaborative management among different surgical teams. Finally, the diversity of techniques employed led us to conduct a descriptive analysis, as meaningful statistical comparisons could not be established.

Finally, a potential selection bias must be acknowledged. The improved outcomes observed in the group undergoing a deferred single-stage approach may, at least in part, reflect an *a priori* selection of patients with a favorable clinical course after the first procedure, who did no develop complications and were therefore eligible for the second intervention. This potential bias limits the generalizability of the results and underscores the need for prospective comparative studies.

## Conclusion

This study demonstrates that it is feasible to plan a two-stage surgical approach for intestinal restoration and abdominal wall reconstruction during the same hospital admission. This strategy allows the distribution of surgical complexity and may reduce postoperative morbidity by separating the interventions. However, a substantial proportion of patients may ultimately not undergo the second surgical stage.

Therefore, future comparative studies between single-stage and two-stage approaches are required to more precisely define the most appropriate indications for each strategy. In addition, further evidence is needed to assess the safety, costs, and clinical implications of performing these interventions either simultaneously or sequentially. This work represents a meaningful step toward generating key evidence to support the development and refinement of this surgical approach.

## Data Availability

The datasets presented in this article are not readily available because the datasets generated and analyzed during the current study are derived from the institutional clinical records and surgical database of Clínica Universitaria Colombia (Bogotá, Colombia). These data were collected prospectively and reviewed retrospectively for this research. Due to privacy and ethical restrictions, individual patient data are not publicly available, but anonymized data may be provided by the corresponding author upon reasonable request and with authorization from the institutional ethics committee. Requests to access the datasets should be directed to coloproctologiacuc@gmail.com.
